# Der p 1 Disrupts the Epithelial Barrier and Induces IL-6 Production in Patients With House Dust Mite Allergic Rhinitis

**DOI:** 10.3389/falgy.2021.692049

**Published:** 2021-08-03

**Authors:** Kazuhiro Ogi, Mahnaz Ramezanpour, Sha Liu, Jannatul Ferdoush Tuli, Catherine Bennett, Masanobu Suzuki, Shigeharu Fujieda, Alkis James Psaltis, Peter-John Wormald, Sarah Vreugde

**Affiliations:** ^1^Department of Surgery–Otolaryngology, Head and Neck Surgery, University of Adelaide, Adelaide, SA, Australia; ^2^Central Adelaide Local Health Network, The Queen Elizabeth Hospital, Basil Hetzel Institute for Translational Health Research, Woodville South, SA, Australia; ^3^Division of Otorhinolaryngology Head and Neck Surgery, Department of Sensory and Locomotor Medicine, Faculty of Medical Sciences, University of Fukui, Fukui, Japan

**Keywords:** Der p 1, claudin-1, primary nasal epithelial cells, allergic rhinitis, house dust mite

## Abstract

**Background:** *Dermatophagoides pteronyssinus* 1/2 (Der p 1/Der p 2) are regarded as important allergens of house dust mite (HDM). However, the effect of both products on the epithelial barrier and immune response of patients with and without HDM allergic rhinitis (AR) remains unclear.

**Methods:** Air–liquid interface (ALI) cultured human nasal epithelial cells (HNECs) derived from control subjects (non-AR) (*n* = 9) and HDM-AR patients (*n* = 9) were treated with Der P 1 and Der P 2, followed by testing the transepithelial electrical resistance (TEER), paracellular permeability of fluorescein isothiocyanate (FITC)-dextrans and immunofluorescence of claudin-1 and ZO-1. Interleukin-6 (IL-6) production was evaluated by ELISA.

**Results:** Der p 1 reduced TEER significantly in a transient and dose-dependent manner in HNEC-ALI cultures from HDM-AR and non-AR patients, whilst the paracellular permeability was not affected. TEER was significantly reduced by Der p 1 at the 10-min time point in HDM-AR patients compared to non-AR patients (*p* = 0.0259). Compared to no-treatment control, in HNECs derived from HDM-AR patients, Der p 1 significantly cleaved claudin-1 after 30 min exposure (72.7 ± 9.5 % in non-AR group, 39.9 ± 7.1 % in HDM-AR group, *p* = 0.0286) and induced IL-6 secretion (*p* = 0.0271).

**Conclusions:** Our results suggest that patients with HDM-AR are more sensitive to Der p 1 than non-AR patients with increased effects of Der p1 on the mucosal barrier and induction of inflammation, indicating an important role for Der p1 in sensitization and HDM-AR development.

## Introduction

Allergic rhinitis (AR) is a common disorder involving immunoglobulin E (IgE)-mediated type I allergic inflammation of the nasal mucosa following allergen exposure (1). Typical AR symptoms are characterized by rhinorrhea, nasal congestion, nasal itching, and sneezing, which can affect the patient's physical and mental health and work performance (2). AR is a multifactorial disease where both genetic factors and environmental factors play a role. The most common environmental factors leading to the development of AR are allergens, and house dust mites (HDM) are the most common aeroallergens worldwide in not only perennial AR but also allergic asthma (3). Perennial AR is frequently accompanied by asthma that can cause severe morbidity and mortality, especially among children (4).

There are more than 20 types of HDM allergens on the list of World Health Organization/International Union of Immunological Societies and more allergens are still being investigated (5). *Dermatophagoides pteronyssinus* 1/2 (Der p 1/Der p 2) are major components of HDM extracts and are regarded as the most important HDM allergens. Der p 1 is a 25-kDa protein and has enzymatic activity as a cysteine protease. It is classified as a digestive tract enzyme as Der p 1 is found mainly in the excrement of mites. Previous studies reported that Der p 1 increased the permeability of airway epithelial cells via its protease activity (6). Der p 2 is a 14-kDa protein, mainly found in the body of mites rather than excrement. Der p 2 has been shown to promote lipopolysaccharide-driven Toll-like receptor (TLR) 4 signaling and T helper cells type 2 (Th2) polarization (7).

The airway epithelium is known to constitute a physical barrier against the penetration of not only pathogens but also numerous allergens, effectively acting as the first line of host defense. Tight junctions (TJs), formed between the apical surface of adjacent cells, are considered to be vital components in maintaining the epithelial cell and mucosal membrane integrity. TJs limit the diffusion of solutes through the intercellular space and provide cellular polarity separating apical and basolateral domains (8, 9). In addition to these “barrier” and “fence” functions, TJs were shown to have an important role in signal transduction involved in regulating the epithelial barrier, cell proliferation and differentiation (10, 11). TJ failure and vulnerability of the epithelial barrier have been reported in patients with asthma (12), AR (13), and atopic dermatitis (14, 15). The clinical relationship between asthma, allergic rhinitis, and atopic dermatitis, the so-called “allergic triad”, is well known and genetic association studies have found genetic alterations in shared pathways of immune regulation and promotion of Th2 cytokine production by epithelial cells (16). The inherited barrier dysfunction and barrier disruption caused by environmental allergens are considered to contribute to the sensitization and the progression of the “atopic march”, where atopic dermatitis is followed by asthma and allergic rhinitis onset (17). There are a large number of reports about the presence of barrier disruption in atopic dermatitis and asthma (12, 18). They have shown various effects of HDM allergens on the skin barrier and lower airway epithelial barrier. However, only few studies have been conducted investigating the effects of HDM on the human nasal mucosal barrier (13, 19, 20).

In order to develop effective treatments for AR it is vital to understand the mechanisms of how allergens affect the nasal mucosa. The aim of this study was to evaluate the effect of Der p 1 and Der p 2 on the barrier of air-liquid interface cultured primary human nasal epithelial cells derived from patients with and without HDM-AR.

## Materials and Methods

### Human Subjects

This study was performed with approval of The Central Adelaide Local Health Network Human Research Ethics Committee (reference HREC/15/TQEH/132 and 13604). All participants provided informed written consent in accordance with the Declaration of Helsinki. Primary human nasal epithelial cells (HNECs) were obtained from the inferior turbinate surface with sterile nasal brushes during paranasal sinus, nasal septum or pituitary tumor surgeries. The diagnosis of HDM allergy was based on the International Consensus Statement on Allergy and Rhinology (1). Patients with house dust mite (HDM) allergy were categorized in the HDM-allergic rhinitis (HDM-AR) group and patients without HDM allergy were categorized as non-AR group. They self-reported a previous clinical diagnosis of HDM-AR either via a positive skin prick test or serum antigen-specific IgE test for house dust mite antigens. All of the HDM-AR patients were AR symptomatic, while patients in the non-AR group did not have a history of AR. Exclusion criteria included chronic rhinosinusitis with nasal polyps, active smoking, systemic disease and the use of systemic steroids. Patient information is shown in Table 1.

**Table 1:** Patient characteristics on TEER, paracellular flux and IL-6 production.

**Item**	**Non-AR**	**HDM-AR**	***p*** **-value**
Subjects for TEER and ELISA	9	9	
Age	52.4 ± 5.5	35.9 ± 2.7	N.S
Male/female	4/5	6/3	N.S
CRSsNP/control	6/3	7/2	N.S
Sensitization			
HDM	0	9	<0.0001
Except for HDM	0	2	N.S
Baseline TEER (Ω × cm^2^)	1463.3 ± 123.7	1607.0 ± 100.2	N.S
Subjects for IF	4	4	
Age	47.3 ± 5.4	40 ± 6.7	N.S
Male/female	2/2	2/2	N.S
CRSsNP/control	0/4	0/4	N.S
Sensitization			
HDM	0	4	<0.05
Except for HDM	0	0	N.S

*The values are shown as numbers or mean ± SEM. The statistical significance was evaluated by Student t test or Fisher's exact test*.

*CRSsNP, chronic rhinosinusitis without nasal polyps; HDM, house dust mite; AR, allergic rhinitis. IF, immunofluorescence; TEER, transepithelial electrical resistance*.

### Cell Culture

The nasal brushes were immediately transferred into Dulbecco's Modified Eagle Medium (Life technologies corporation, Grand Island, NY, USA), then HNECs were collected after centrifugation (500 g for 7 min) and resuspended in Ex-medium consisting of PneumaCult™-Ex Plus Basal Medium (STEMCELL Technologies, Tullamarine, VIC, Australia) along with PneumaCult™-Ex Plus 50X Supplement, Hydrocortisone Stock Solution (STEMCELL, Vancouver, Canada) and penicillin-streptomycin/amphotericin B (Thermo Scientific, Walthman, MA, USA). The resuspended cells were incubated in culture plates coated with anti-CD68 (Dako, Glostrup, Denmark) to remove macrophages for 20 min at 37°C. Supernatants containing HNECs were seeded in collagen-coated T75 cell culture flasks (Corning Incorporated, NY, USA) and grown in Ex-medium at 37°C, 5% CO_2_, 95% humidity. Once the cells were 80% confluent, they were washed with phosphate buffered saline (PBS) and incubated with 0.05% trypsin (Thermo Scientific, Waltham, MA, USA). After 5 min the trypsin reaction was neutralized with 10% fetal bovine serum in PBS, the cells were centrifuged (5 min, 400 g) and resuspended in Ex medium. Cell suspensions (70,000 cells) were seeded in 100 μL Ex medium onto collagen coated apical chambers of 6.5-mm-diameter polyester membranes with a pore size of 0.4 μm (Corning Incorporated, NY, USA). 500 μL Ex medium was added in the basolateral chamber. After 2 days, the medium was removed from the apical chamber and the basolateral chamber medium was changed to ALI medium to allow differentiation of cells. ALI medium consisted of PneumaCult™-ALI Basal Medium (STEMCELL Technologies, Tullamarine, VIC, Australia) along with PneumaCult™-ALI 10X Supplement, PneumaCult™-ALI Maintenance Supplement (STEMCELL, Vancouver, Canada) and penicillin-streptomycin/amphotericin B (Thermo Scientific, Walthman, MA, USA). Medium was changed every 48–72 h. The cells were cultured for at least 21 days before conducting any experiment (21).

### Measurement of Transepithelial Electrical Resistance (TEER)

ALI cultured HNECs were treated with purified protein Der P 1 or Der P 2 (purity of 98%) (Citeq Biologics, Groningen, The Netherlands) at 0.4, 2, and 4 mM concentrations (diluted in PBS) and TEER was measured with a voltohmmeter at various time points up to 6 h (EVOM, World Precision Instruments, Sarasota, FL, USA) whilst putting cell cultures on a heating plate (LEC Instrument, Australia). PBS was added as a negative control and 5% Triton was added as a positive control. TEER measurements at each time point were normalized with the values at time = 0 before treatments exposure (22).

### Paracellular Flux Measurement

To measure the paracellular flux, 4-kDa fluorescein isothiocyanate (FITC)-dextran (Sigma-Aldrich, St. Louis, MO, USA) was used as a tracer. FITC-dextran was diluted in PBS at a concentration of 3 mg/ml. The apical chamber of HNEC-ALI cultures was washed two times with PBS after the last (6 h) TEER measurement followed by incubation with 100 μL FITC-dextran at 37°C, 5% CO_2_, 95% humidity for 2 h. Samples were then taken from the basolateral compartment and transferred to a clear bottom black 96-well plate. The amount of passaged FITC-dextran was measured by a FLUOstar Optima 96-well fluorescence microplate reader (BMG Labtech, Ortenberg, Germany) at excitation and emission wavelengths of 485 and 520 nm, respectively.

### Immunofluorescence Staining

HNEC-ALI were fixed in PBS containing 2.5 % formalin for 10 min and preserved at −20°C. HNEC-ALI were permeabilized with 1% sodium dodecyl sulfate on ice for 15 min. Permeabilized cells were then blocked with serum free blocker (Dako, Glostrup, Denmark) at room temperature for 1 h, followed by overnight incubation with primary antibodies diluted in PBS: 1:50 rabbit anti-claudin-1; 1:100 mouse anti-zonula occludens (ZO)-1 (Invitrogen, Carlsbad, CA, USA). This was followed by incubation of donkey anti-rabbit Cy3 (1:200 in PBS) and donkey anti-mouse IgG Alexa Fluor 488 (Jackson ImmunoResearch Labs Inc., West Grove, PA, USA; 1:200 diluted in PBS) and incubated for 1 h at room temperature. Then nuclei were stained by incubation with 200 ng/ml of 4′,6-diamidino-2-phenylindole (DAPI; Sigma-Aldrich, St. Louis, MO, USA) for 10 min at room temperature. Finally, HNEC-ALI cultures were mounted with a drop of fluorescence anti-fade mounting medium (Dako, Glostrup, Denmark) before cover slipping. The samples were washed with PBS four times between each step. Images were recorded using a confocal laser-scanning microscope LSM700 (Zeiss Microscopy, Jena, Germany) with × 20 magnification. Image processing was performed with ZEN Imaging Software (Carl Zeiss AG, Oberkochen, Germany). Three different areas were randomly selected for quantification in individual samples. Digital image stacks were created as 20 μm thickness with 21 sequential slices. The mean fluorescence intensity of claudin-1 and ZO-1 was normalized against DAPI.

### IL-6 ELISA Assay

Interleukin-6 (IL-6) levels produced by HNEC-ALI cultures were measured with enzyme-linked immunosorbent assay (ELISA) kit (BD Biosciences, Franklin Lakes, NJ, USA) after application of 4 mM Der p 1, 4 mM Der p 2 for 6 h. PBS was used as negative control and 10 μg/ml poly (I:C) LMW (Invivogen, San Diego, CA, USA) was used as positive control. The medium in the basolateral chamber was harvested after completion of the TEER experiment and stored at −20°C. Amounts of IL-6 in the medium were determined according to the manufacturers' protocols. All samples were measured in duplicate. The optical density (OD) was measured at 450 nm and IL-6 concentration was determined using the standard curve prepared for individual assay (21, 23, 24).

### Statistical Analysis

All results are presented as the mean ± standard error of the mean (SEM). The collected data were analyzed with statistical software (Graph Pad Prism 7, San Diego, CA, USA). The comparisons in multiple treatment conditions were conducted by one-way analysis of variance (ANOVA), followed by a Dunnett's test. Statistical significance between two groups was determined by using a 2-tailed unpaired *t* test or Fisher exact test. *P* < 0.05 were considered statistically significant.

## Results

### Der p 1 Attenuates Transepithelial Electrical Resistance (TEER) in HNEC-ALI Cultures

**Figure 1:**
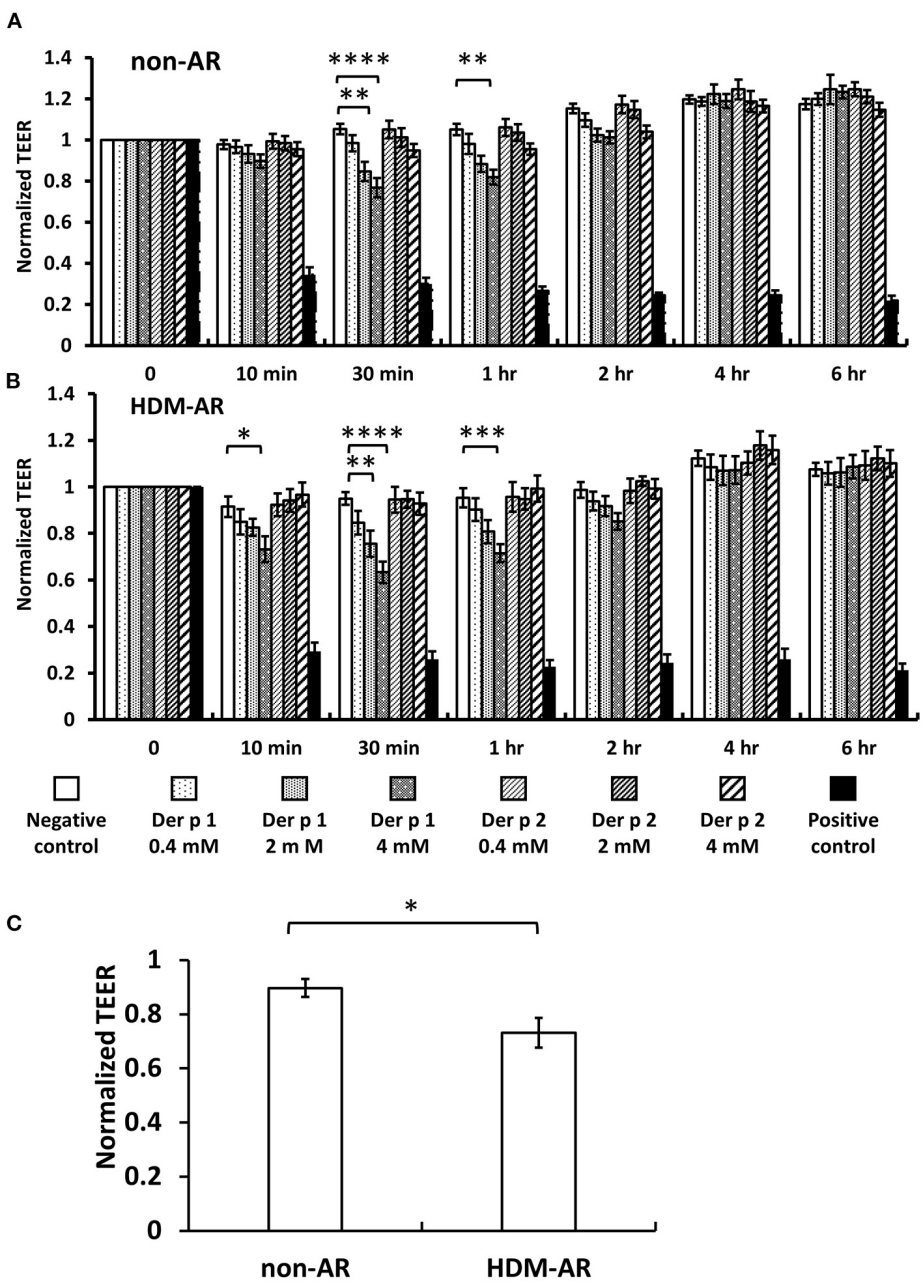
Der p 1 reduced TEER of HNEC-ALI cultures transiently in a dose dependent manner. TEER was measured at time point = 0 (immediately after application of test compound), 10, 30 min, 1 h and hourly for 6 h after application of PBS (negative control), 0.4, 2, and 4 mM Derp1 or Derp2 or 5% Triton (positive control) to HNEC-ALI cultures from non-AR patients **(A)** or HDM-AR patients **(B)**. Values were normalized to the values at time point = 0 and compared to negative control at the same time point. *p < 0.05, **p < 0.01, ***p < 0.001, ****p < 0.0001, one-way ANOVA followed by Dunnett's test. **(C)** TEER reduction by 4 mM Der p 1 challenge for 10 min was compared between non-AR group and HDM-AR group. *p < 0.05, Student *t* test. HDM, house dust mite; AR, allergic rhinitis All data are shown as the means ± SEM. (*n* = 9; respectively for each group).

**Figure 2:**
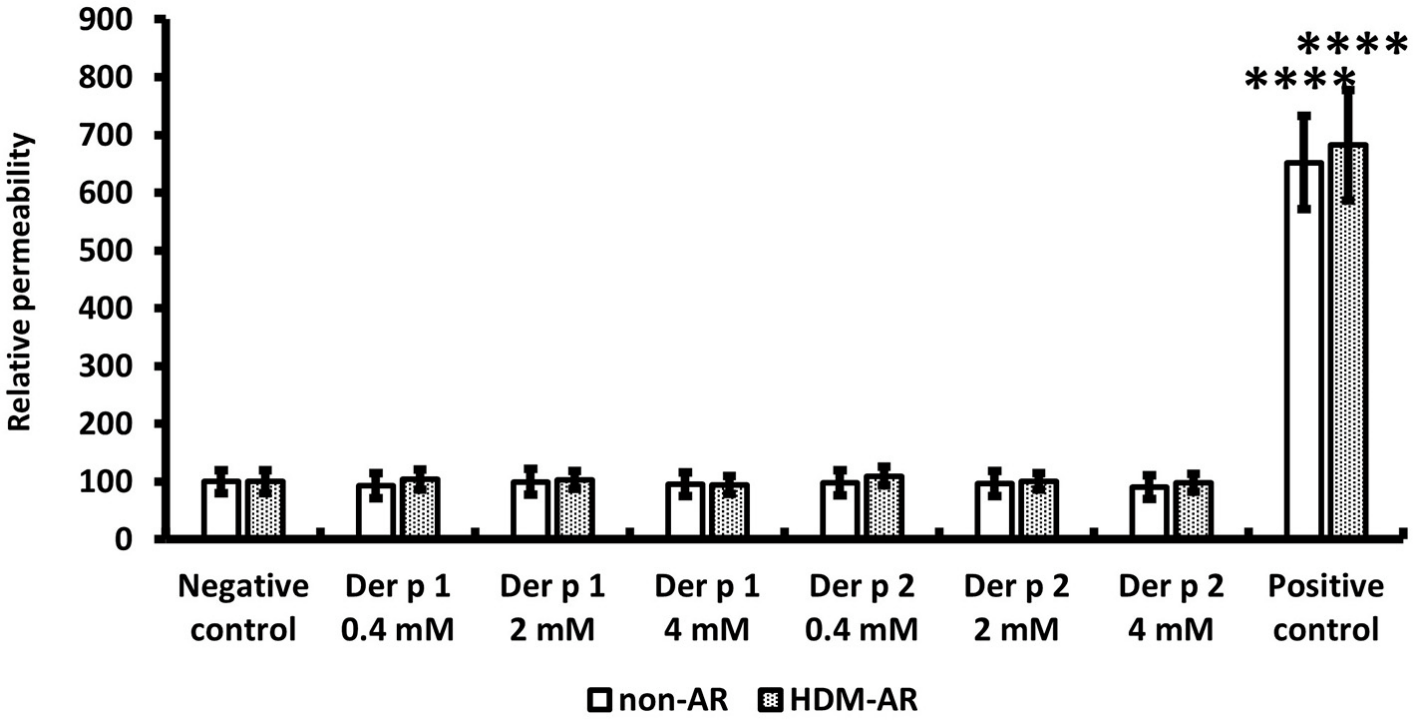
Paracellular permeability was not affected by HDM extracts. FITC-dextran passage was measured in each treated sample after 6 h TEER measurement and normalized to the negative control. PBS was used as the negative control and 5% Triton was used as the positive control. The data is shown as the means ± SEM. (*n* = 9; respectively for each group). ****p < 0.0001, one-way ANOVA followed by Dunnett's test.

HNEC-ALI cultures were established from 18 subjects (HDM-AR, *n* = 9; non-AR, *n* = 9). Demographic factors and comorbidities of patients are detailed in Table 1. There was no significant difference in TEER measured at baseline between HDM-AR and non-AR groups (*p* = 0.30) (Table 1). In HNEC-ALI cultures derived from both HDM-AR and non-AR patients, Der p 1 affected TEER in a transient, dose- and time-dependent manner with a significant reduction in TEER in Der p 1 challenged cells compared to control at the 30-min (*p* = 0.0021 for 2 mM and *p* < 0.0001 for 4 mM in non-AR group; *p* = 0.0085 for 2mM and *p* < 0.0001 for 4mM in HDM-AR group) and 1-h time point (*p* = 0.0031 for 4mM in non-AR group; *p* = 0.0030 for 4mM in HDM-AR group) (Figures 1A,B). The maximum TEER reduction was seen after 30 min of challenge (*p* < 0.0001 in both groups), followed by a gradual restoration of TEER back to baseline from the 2-h time point onwards. A significant reduction in TEER was observed in HNEC-ALI cultures treated with 4 mM Der p 1 at the 10-min time point in cells derived from HDM-AR patients (*p* = 0.0321) but not in cells derived from non-AR patients (*p* = 0.1136). TEER values were significantly reduced upon challenge with 4 mM Der p 1 at the 10-min time point in HDM-AR patients compared to non-AR patients (*p* = 0.0259, Figure 1C). In contrast, Der p 2 had no effect on TEER in any of the groups. As shown in Figure 2, the paracellular permeability was not affected by Der p 1 nor Der p 2 after 6 h incubation in both groups (*p* > 0.05).

### Der p 1 Exposure Results in a Transient Cleavage of Claudin-1 but Not ZO-1 in HDM-AR Group

**Figure 3:**
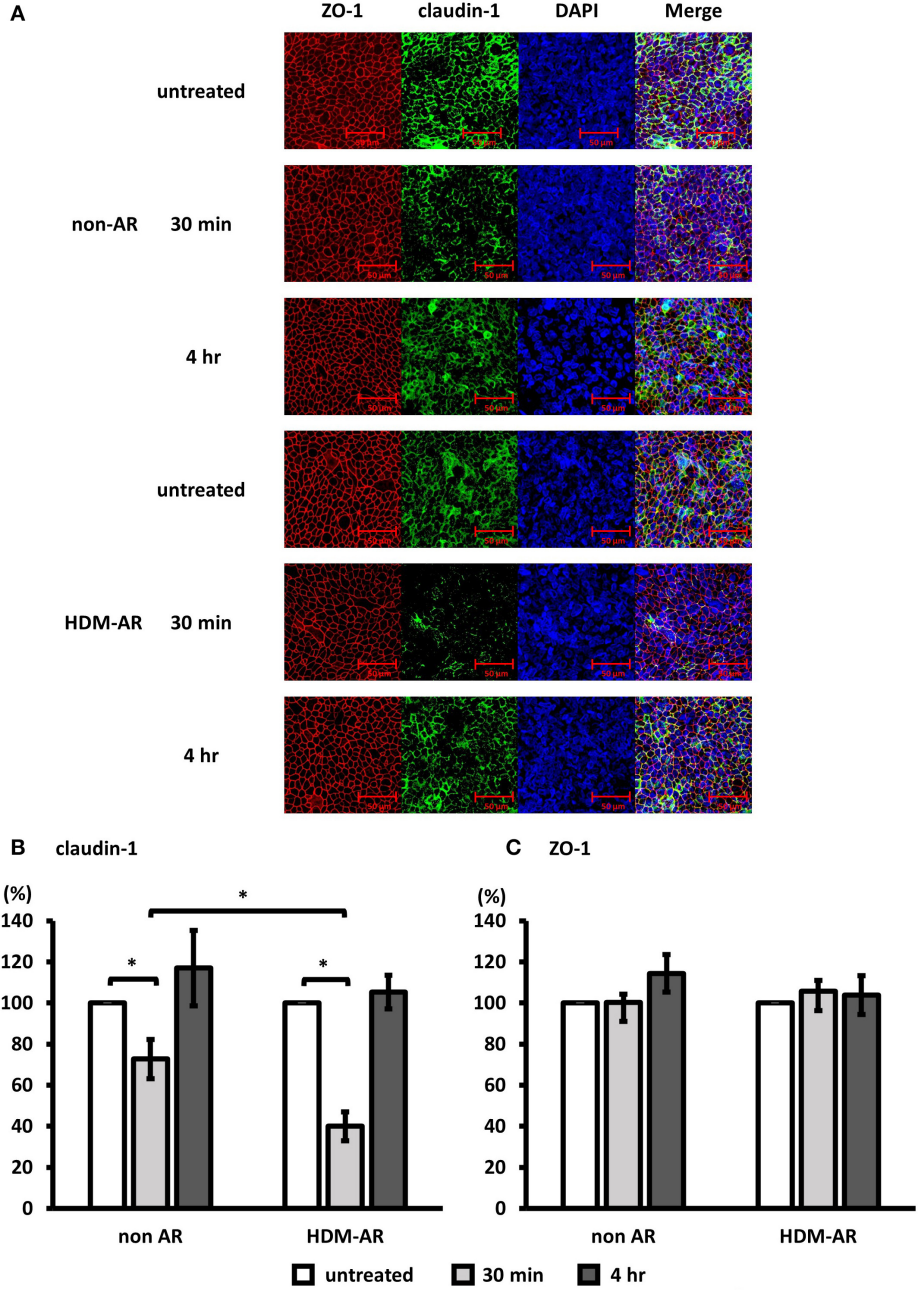
Der p 1 disrupted claudin-1 expression transiently in HNEC-ALI cultures. Representative immunofluorescence images of HNEC-ALI cells from non-AR patients and HDM-AR patients double stained with claudin-1 **(A,B)** and ZO-1 **(A,C)** antibodies after challenge with PBS (untreated) or 4 mM Der p 1 for 30 min and 4 h. Red, ZO-1; green, claudin-1; blue, DAPI. Magnification is 20 × **(A)**. Fluorescence intensity of claudin-1 **(B)** and ZO-1 **(C)** expression normalized with DAPI after 30 min and 4 h exposure with or without 4 mM Der p 1. The data is shown as the means ± SEM. (*n* = 4; respectively for each group). *p < 0.05, one-way ANOVA followed by Dunnett's test.

Next, we examined the effect of Der p 1 challenge of HNEC-ALI cells on the immunolocalisation of the TJ proteins claudin-1 and ZO-1. HNEC-ALI cultures were established from eight donors (non-AR; *n* = 4, HDM-AR; *n* = 4) (Table 1) and treated with 4 mM Der p 1 for 30 min and 4 h. Der p 1 significantly induced cleavage of claudin-1 in both groups after 30 min exposure compared to no-treatment control (72.7 ± 9.5 % in non-AR group, 39.9 ± 7.1 % in HDM-AR group) (Figures 3A,B). Moreover, the claudin-1 cleavage after 30 min incubation with Der p 1 was significant in HNEC-ALI cultures derived from HDM-AR patients compared to HNEC-ALI cultures derived from non-AR patients (*p* = 0.0286). Similar to the transient TEER change, the claudin-1 localization normalized at the 4 h time point in both groups. The localization of ZO-1 was not altered by challenge with Der p 1 (*p* > 0.05) (Figures 3A,C).

### Der p 1 Exposure Induces IL-6 Secretion in HNEC-ALI Cultures of HDM-AR Patients

**Figure 4:**
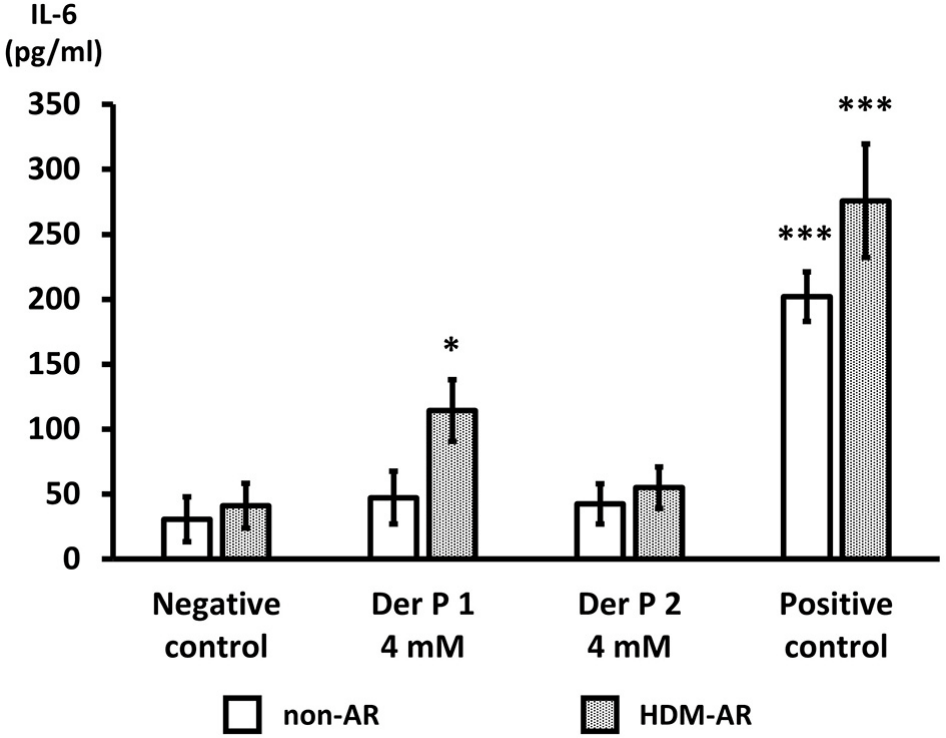
Der p 1 exposure induces IL-6 secretion in HNEC-ALI cultures of HDM-AR patients. IL-6 production (pg/ml) after 6-h exposure to PBS (negative control), Der p 1 (4 mM), Der p 2 (4 mM) and Poly (I:C) LMW (10 μg/ml) (positive control) to HNEC-ALI cultures from non-AR or HDM-AR patients. The data is shown as the means ± SEM. (*n* = 9; respectively for each group). *p < 0.05, ***p < 0.001, one-way ANOVA followed by Dunnett's test.

Next, we examined IL-6 production from HNEC-ALI cells treated with Der p 1 and Der p 2. PBS (negative control) and 10 μg/ml poly (I:C) LMW were used as negative and positive control respectively. Compared to negative control, Der p 1 induced IL-6 secretion in cells derived from HDM-AR patients (*p* = 0.0271) but not in cells derived from non-AR patients. Der p 2 did not significantly induce IL-6 production in cells derived from HDM-AR or non-AR patients (Figure 4).

## Discussion

Our study demonstrates a dose-dependent transient Der p 1-induced detrimental effect on the nasal epithelial barrier in ALI cultured HNECs associated with a cleavage of claudin-1. The effect was significantly higher in patients with HDM-AR compared to non-AR patients and was accompanied by a higher IL-6 production in those patients. These results suggest that the epithelium of patients with HDM-AR is more sensitive to Der p 1 than that from patients without AR. This may possibly create a higher chance of sensitization to Der p 1 in these patients with mucosal barrier disruption and induction of inflammation.

Previous research has shown an impaired nasal epithelial barrier function in patients with HDM-induced AR, indicated by a low TEER and low mRNA expression of TJ genes compared to control (13). Whilst our study could not identify differences in baseline TEER measures in HNEC-ALI cultures derived from HDM-AR patients compared to non-AR patients, it did show differences between those two patient cohorts in their response to Der p 1. Our results therefore support the hypothesis that HDM-AR patients might be more sensitive to challenges with Der p1 resulting in impaired barrier function.

Interestingly, our data showed that the Der p 1-dependent effect on TEER was accompanied by a reduced expression of claudin-1 but not ZO-1. Together with the finding that paracellular permeability of larger molecules was not affected, these findings suggest that Der p1 affects mainly the pore pathway and does not promote an increased paracellular flux of larger non-charged solutes (25). Given the mass of Der p1 is approximately 25 kDa, this data implies that Der p 1 alone is unlikely to facilitate the penetration of Der p1 or other larger molecules into the mucosa by enhanced paracellular transport. Results of a recent study suggests, however, that epithelial barrier disruption results in enhanced sensitization and mast cell degranulation in response to ovalbumin exposure even in non-inflammatory conditions *in vivo* (26). It is therefore considered this disruptive effect of Der p 1 on the mucosal barrier contributes to sensitization to allergen, even if enhanced penetration of allergens and other large molecules within the mucosa is unlikely. One potential mechanism might involve dendritic cells. Those cells express several TJ proteins such as claudin-1,−7 and occludin, that interact with TJ proteins of epithelial cells with dendrites penetrating beyond the apical mucosal surface within the lumen of the sinonasal cavity in patients with AR and it could be that Der p1 affects this process (27–30). Interestingly, Der p 1 has also been shown to not only directly disrupt TJ proteins, it can also act upon a cell surface zymogen resulting trans-epithelial delivery of allergen (20). Further research is needed to determine the potential role of these different contributing factors and cells to allergen sensitization and development of HDM-AR.

Our study is the first to show recovery of TEER and claudin-1 protein expression of HNEC-ALI cultured cells after Der p-1 exposure. These results are in line with other studies showing a reversible effect of Der p1 on the mucosal barrier (20). Conversely, there are several reports indicating a persistent reduction of TEER and increased paracellular permeability by Der p 1 (19, 31, 32). These apparent discrepancies could be related to differences in the purity and concentration of HDM extracts, cell types and experimental conditions. Alternatively, it might be that the enzymatic activity of Der p 1 was inactivated during the experimental procedure even though the cells and medium were kept in physiological condition to mimic airway exposure. A limitation of the study is that the Der p 1 has not been pretreated with reducing agents to restore the full protease activity (33). Further studies are required with pretreated purified extracts to maximize their activity and to evaluate how that affects their barrier disruptive effects. Regardless, our results suggest that epithelial barrier structure and function after Der p1 exposure is controlled dynamically and supports the notion of a remarkable plasticity under various physiological and pathological conditions (8, 34). Although the molecular mechanism of Der p1 induced barrier dysfunction in HNECs remains unknown, several signaling pathways, such as serine/threonine/tyrosine phosphorylation and small G proteins and protein kinase C signaling are thought to be implicated (35, 36).

Claudin-1 is expressed in the paracellular space, while ZO-1 is an intracellular molecule (25, 35). There are several studies reporting destructive effects of Der p 1 on ZO-1 and occludin, though relatively fewer studies on the expression of claudins are available (13, 19, 31). We studied claudin-1 because it is expressed in all airway epithelial cells and is regarded as representative of the claudin family of proteins which have a “sealing function” (37). Moreover, claudin-1 possesses a putative cleavage site by Der p 1 in its first extracellular domain (20, 38, 39). Previous studies reported no difference in mRNA expression of claudin-1 between patients with HDM-AR and a control group (13). Although we also did not find a difference in the baseline protein expression of claudin-1 between HDM-AR group and non-AR control group, claudin-1 expression was significantly reduced after Der p 1 challenge in the HDM-AR group compared to the non-AR patient group. Together, these results suggest that HDM-AR patients are more sensitive to Der p1 than non-AR patients and that cleavage of claudin-1 might occur by a direct action on the trans-cellular protein domain. Further studies are needed to confirm this hypothesis and to determine the molecular base of enhanced sensitivity to Der p1 in HDM-AR patients. Also, given that more than 20 types of claudins have been identified, it would be interesting to define the expression of the different claudin family members in HNEC-ALI cultures and to evaluate the effect of Der p1 on each of those claudins (40, 41). It is known that some people have no symptoms of HDM-AR regardless of showing sensitization evidence called non-progressors (42). This sensitivity difference of HDM-AR derived HNEC-ALI cultures and claudin-1 upon challenge with Der p 1 could relate to the potential risk of developing AR.

We studied the IL-6 production because previous studies indicated the relationship between the dysregulation of TJ protein expression and levels of inflammatory cytokines such as IL-6 and tumor necrosis factor (43, 44). Der p 1 has been shown to activate protease-activated receptor (PAR)-2 and induces proinflammatory cytokines expression by epithelial cells (45). Moreover, TLR4 is also activated by Der p 1 (46) and Der p 1's protease activity could contribute to IL-6 production by a PAR independent mechanism (47). Alternatively, the mechanism of IL-6 production observed in our study in response to Der p1 might involve the PAR/PI3K/NFκB signaling pathway (47, 48). However, the relationship between Der p 1 and PAR activation and the mechanism of enhanced IL-6 production by HNECs derived from HDM-AR patients, as seen in our study, is unknown and requires further investigation (49, 50). Although we assumed that Der p 2 would induce IL-6 production as it promotes TLR4 signaling, there was no significant IL-6 production in both groups (7). Other reports also found no effect of Der p 2 on cytokine production (47). It would be interesting to test the synergistic effects of lipopolysaccharide (LPS) and Der p 2, because Der p 2 facilitates LPS-driven TLR4 signaling (7).

In summary, Der p 1 induces a reversible degradation of claudin-1 in HNEC-ALI cultures derived from HDM-AR patients with a reduced mucosal barrier structure and function and accompanied by an induction of IL-6 secretion. Our data therefore support an important role for Der p1 in sensitization and AR development.

## Data Availability Statement

The raw data supporting the conclusions of this article will be made available by the authors, without undue reservation.

## Ethics Statement

The studies involving human participants were reviewed and approved by The Central Adelaide Local Health Network Human Research Ethics Committee. The patients/participants provided their written informed consent to participate in this study.

## Author Contributions

KO, JF, and MR conducted the biological experiments. CB, AP, and P-JW collected the clinical samples. SL, AP, P-JW, and SV designed the study. MS, MR, and SV analyzed the data. SF, KO, and SV drafted the manuscript. All authors critically reviewed the manuscript and approved the final version.

## Conflict of Interest

The authors declare that the research was conducted in the absence of any commercial or financial relationships that could be construed as a potential conflict of interest.

## Publisher's Note

All claims expressed in this article are solely those of the authors and do not necessarily represent those of their affiliated organizations, or those of the publisher, the editors and the reviewers. Any product that may be evaluated in this article, or claim that may be made by its manufacturer, is not guaranteed or endorsed by the publisher.

## Abbreviations

ALI: air-liquid interface
ANOVA: one-way analysis of variance
AR: allergic rhinitis
DAPI: 4′,6-diamidino-2-phenylindole
Der p: *Dermatophagoides pteronyssinus*
ELISA: enzyme-linked immunosorbent assay
FITC: fluorescein isothiocyanate
HDM: house dust mite
HNEC: human nasal epithelial cell
IgE: immunoglobulin E
IL: interleukin
PBS: phosphate buffered saline
PAR: protease-activated receptor
SEM: standard error of the mean
TEER: transepithelial electric resistance
Th2: T helper cells type 2
TJ: tight junction
TLR: Toll-like receptor
ZO: zonula occludens.
